# Effect of comprehensive geriatric assessment for frail elderly patients operated for colorectal cancer—the colorectal cancer frailty study: study protocol for a randomized, controlled, multicentre trial

**DOI:** 10.1186/s13063-022-06883-9

**Published:** 2022-11-17

**Authors:** Maria Normann, Niklas Ekerstad, Eva Angenete, Mattias Prytz

**Affiliations:** 1 Department of Surgery, Institute of Clinical Sciences, Sahlgrenska Academy, University of Gothenburg, Gothenburg, Sweden; 2 Department of Surgery, Region Västra Götaland, NU-Hospital Group, Trollhättan, Sweden; 3 Department of Health, Medicine and Caring Sciences, Linköping University, Linköping, Sweden; 4 Department of Research and Development, Region Västra Götaland, NU-Hospital Group, Trollhättan, Sweden; 5 Department of Surgery, SSORG – Scandinavian Surgical Outcomes Research Group, Institute of Clinical Sciences, Sahlgrenska Academy, University of Gothenburg, Gothenburg, Sweden; 6 Department of Surgery, Region Västra Götaland, Sahlgrenska University Hospital, Gothenburg, Sweden

**Keywords:** CGA and care, Colorectal neoplasm, Elderly, ERAS, Frailty, PICO (patient-intervention-comparison-outcome), Surgery, RCT (randomised controlled trial)

## Abstract

**Background:**

Colorectal cancer (CRC) is the third most common cancer worldwide, with a median age of 72–75 years at diagnosis. Curative treatment usually involves surgery; if left untreated, symptoms may require emergency surgery. Therefore, most patients will be accepted for surgery, despite of high age or comorbidity. It is known that elderly patients suffer higher risks after surgery than younger patients, in terms of complications and mortality. Assessing frailty and offering frail elderly patients individualized treatment according to the comprehensive geriatric assessment (CGA) and care concept has been shown to improve the outcome for frail elderly patients in other clinical contexts.

**Methods:**

This randomized controlled multicentre trial aims to investigate if CGA and care prior to curatively intended surgery for CRC in frail elderly patients will improve postoperative outcome. All patients ≥ 70 years with surgically curable CRC will be screened for frailty using the Clinical Frailty Scale (CFS-9). Frail patients will be offered inclusion. Randomization is stratified for colon or rectal cancer. Patients in the intervention group are, in addition to standard protocol, treated according to CGA and care. This consists of individualized assessments and interventions, established by a multiprofessional team. Patients in the control group are treated according to best known practice as stipulated by Swedish colorectal cancer treatment guidelines, within an enhanced recovery after surgery (ERAS) setting. The primary outcome is 90-day mortality. Secondary outcomes are the length of hospital stay and total number of hospital days within 3 months, discharge destination, 30-day readmission, ADL, safe medication assessment, CFS-9 score, complications, Health-Related Quality of Life (HRQoL) at 2-month follow-up in comparison to baseline measurements, health economical calculations including cost-effectiveness analysis based on costs of hospital care and primary care, mortality and HRQoL at baseline, 2- and 12-month follow-up and all-cause 1-year mortality.

**Discussion:**

The trial is the first of its size and extent to investigate intervention with CGA and care prior to surgery for CRC in frail elderly patients. If this addition proves to be favourable, it could have implications on future care of frail elderly patients with CRC.

**Trial registration:**

ClinicalTrials.gov NCT04358328. Registered on 4 February 2020

**Supplementary Information:**

The online version contains supplementary material available at 10.1186/s13063-022-06883-9.

## Administrative information

Note: the numbers in curly brackets in this protocol refer to SPIRIT checklist item numbers. The order of the items has been modified to group similar items (see http://www.equator-network.org/reporting-guidelines/spirit-2013-statement-defining-standard-protocol-items-for-clinical-trials/).

**Table:** 

Title {1}	Effect of Comprehensive Geriatric Assessment (CGA) for frail elderly patients operated for colorectal cancer – The Colorectal Cancer Frailty study: a study protocol for a randomized controlled multicenter trial
Trial registration {2a and 2b}.	ClinicalTrials.gov ID: NCT04358328, 04/02/2020.
Protocol version {3}	June 2021, version 8.1
Funding {4}	Department of Research and Development Centre Västra Götalandsreionen, 2020-12-18, 338 000 SEK (**VGFOUREG-940671).** Department of Research and Development Centre Fyrbodal 2020-05-31, 75 000 SEK (VGFOUFBD-937668). Department of Research and Development Centre Västra Götalandsregionen 2019-12-17, 233 000 SEK (VGFOUREG-931040). Lions’ Cancer Research Fund of Western Sweden, 110 000 SEK.
Author details {5a}	Maria Normann, MD. 1. Department of Surgery, Institute of Clinical Sciences, Sahlgrenska Academy, University of Gothenburg, Gothenburg, Sweden. 2. Region Västra Götaland, NU-Hospital group, Department of Surgery, Trollhättan, Sweden. maria.normann@vgregion.se**Corresponding author.** Niklas Ekerstad, MD, PhD, Associate Professor. 1. Department of Health, Medicine and Caring Sciences, Linköping University, Linköping, Sweden. 2. Region Västra Götaland, NU-Hospital Group, Department of Research and Development, Trollhättan, Sweden. niklas.ekerstad@vgregion.se. Eva Angenete, MD, PhD, Professor of Surgery. 1. Department of Surgery, SSORG – Scandinavian Surgical Outcomes Research Group, Institute of Clinical Sciences, Sahlgrenska Academy, University of Gothenburg, Gothenburg, Sweden. 2. Region Västra Götaland, Sahlgrenska University Hospital, Department of Surgery, Gothenburg, Sweden. eva.angenete@vgregion.se. Mattias Prytz, MD, PhD. 1. Department of Surgery, Institute of Clinical Sciences, Sahlgrenska Academy, University of Gothenburg, Gothenburg, Sweden. 2. Region Västra Götaland, NU-hospital group, Department of Research and Development, Department of Surgery, Trollhättan, Sweden. mattias.prytz@vgregion.se
Name and contact information for the trial sponsor {5b}	The trial sponsors are Region Västra Götaland and the Departments of Surgery at NU-hospital group and at Sahlgrenska University Hospital, and The Research and Development Unit, NU-hospital Group, Sweden.
Role of sponsor {5c}	The trial sponsor has a regulatory role.

## Introduction

### Background and rationale {6a}

Colorectal cancer (CRC) is the third most common cancer form worldwide. Curative treatment involves surgical excision, in many cases in combination with chemo- and/or radiation therapy [1]. The incidence of CRC increases with higher age [1, 2]. In Sweden, the median age of diagnosis of colon cancer is 75 years and the highest number of new cases is in the age group 80–84 years. In terms of rectal cancer, the median age of diagnosis is 72 [3, 4]. As well as age being a risk factor for developing colorectal cancer, it is also known that mortality rates after treatment are higher in the elderly compared to younger CRC patients [5, 6]. Especially the postoperative morbidity and mortality in the first year after surgery are higher in the elderly [7, 8]. The population in Sweden, as in most of the western world, is getting older [9, 10]. This has resulted in elderly patients receiving curatively intended surgery in a larger extent than before [8].

The enhanced recovery after surgery (ERAS) protocol is a multimodal concept covering the pre-, peri-, and postoperative phases striving towards raising the quality of postoperative recovery. Compliance with the ERAS guidelines in colorectal surgery results in a shorter length of hospital stay and in fewer complications and readmissions post-surgery and has thus established its existence in modern colorectal cancer surgery [11].

Research has been conducted to identify elderly patients of increased risk for complications in relation to surgery [6, 12–15]. Chronological age is a blunt way of assessing an individual’s risk, since there is a great diversity in both physical and mental capacity in older people, making the elderly a heterogenous group. Sometimes the term biological age is used to emphasize this fact [16]. The term frailty is recognized as a clinical syndrome of reduced reserves and increased vulnerability and can be used as a marker of biological age [17, 18]. There is an association between frailty and other medical conditions, ageing and degree of disability, but frailty has been established as an independent risk factor for death, decreased ADL-function and hospitalization amongst others [19]. The assessment of frailty enables the identification of elderly individuals in particular risk of adverse health effects [20, 21]. Multiple studies have shown that elderly frail patients suffer a higher risk of severe postoperative complications and mortality compared to elderly non-frail patients after elective surgery for colon and rectal cancer [6, 13, 14, 22–24]. Frailty has even been described as the most important predictor of 90-day mortality post-colon cancer surgery in elderly patients [6] and it correlates to increased hospital and overall health care costs post-colorectal cancer surgery [25].

Two main models are frequently used to describe the frailty syndrome, i.e. the accumulation of deficits model [17] and the frailty phenotype model [18]. The accumulation of deficits model encompasses an assortment of accumulated symptoms, impairments, diseases and disabilities. Fried’s frailty phenotype uses criteria as unintentional weight loss, muscle weakness, physical slowness, poor endurance and low physical activity. There are several different instruments for frailty assessment. The Canadian Study of Health and Ageing (CSHA) Clinical Frailty Scale (CFS-9) is one of the most commonly used frailty instruments in clinical practice [17, 19], and it is validated and easily applied [21–23]. It is based on the accumulation of deficits model and includes assessments of comorbidity, function and cognition and generates a frailty score which describes the patient’s level of frailty [26, 27].

Having targeted the group of frail elderly as high-risk patients, there is a need to gain knowledge on how this group could be treated and further optimized prior to surgery to improve outcomes [8, 22, 28, 29]. Comprehensive geriatric assessment (CGA) and care is a multidimensional, interdisciplinary diagnostic and therapeutic concept. The CGA and care concept has been shown to improve outcomes in frail elderly patients in other clinical contexts [30–32], for example in terms of hip fractures and vascular surgery [33, 34]. A previously published report has investigated CGA and care prior to CRC surgery but in a small context and with short intervention time, without being able to prove any advantages [28]. There is a current discussion on how to ensure adequate treatment of the elderly colorectal cancer patient and a need of consensus on how to balance the risk of discrimination based on chronological age on the one hand and overtreating patients relative their degree of frailty on the other hand. Evidence of a potential benefit of an individualized geriatric treatment in this context has not yet been established. Therefore, there is a need of a randomized controlled trial to further evaluate the supposed advantages of adding CGA and care to standard protocol for the treatment of frail, elderly colorectal cancer patients.

### Objectives {7}

This manuscript is a detailed description of the study design and methodology of the randomized controlled trial “The CRC Frailty study”. The study aims to evaluate if the addition of CGA and care prior to colorectal surgery in the frail elderly could improve postoperative outcome, compared to frail elderly receiving standard care, both within an enhanced recovery after surgery (ERAS) setting.

The primary hypothesis of the RCT is that preoperative intervention with CGA and care, in addition to the conventional pre-, peri-, and postoperative care, will improve 90-day mortality rates after surgery for colon and rectal cancer. Based on previous studies and data from the Swedish Colorectal Cancer Registry (SCRCR) (adjusted for age and ASA classification) [12–14, 35], we know that this group of patients suffer higher mortality rates than younger, robust patients, thus making it highly interesting to investigate whether our intervention can improve this hard endpoint. The secondary hypothesis is that the intervention will improve outcome and recovery after surgery measured as complications, specified in the secondary endpoints stated below.

### Trial design {8}

The study is a prospective, randomized, multicentre superiority trial conducted at departments of surgery performing colorectal cancer surgery in an ERAS setting, in Sweden. Patients will be randomized into two parallel groups: control and intervention. The patients in the intervention group will be treated according to CGA and care preoperatively by geriatric, nursing, physiotherapist and dietician assessments followed by targeted and individualized interventions. The patients in the control group will be handled according to conventional preoperative assessments by anaesthesiologist and standard ERAS care. The groups will be stratified for colon or rectal cancer.

## Methods: participants, interventions and outcomes

### Study setting {9}

The trial is conducted at county and university hospitals in Sweden. A list of participating centres and study sites can be obtained from ClinicalTrials.gov, https://clinicaltrials.gov/ct2/show/NCT04358328. At present, two centres are participating (a county hospital, the Department of Surgery, Norra Älvsborg Hospital (NÄL), Trollhättan, and a tertiary university hospital, the Sahlgrenska University Hospital, Gothenburg) and recruitment of 1–3 further centres is on-going. Potentially available participating centres are departments performing rectal and/or colon cancer surgery according to the ERAS concept, in Region Västra Götaland, Sweden. All levels of hospitals are welcome as this will improve the external validity.

### Eligibility criteria {10}

Inclusion criteria:

- Potentially curable colorectal cancer (according to cTNM)
- Age ≥ 70
- Frailty (defined as CFS-9 score of 4–8 (v2.0))

Exclusion criteria:

- Palliative situation
- Patient unable to understand study information
- Urgent/emergent surgery
- Terminally ill patient (CFS-9 score of 9)
- Estimated life expectancy < 6 months
- Unwillingness to take part in the study

Participating study centres are performing colorectal cancer surgery and applying an ERAS protocol. Participating centres must also be able to supply a multiprofessional CGA and care team consisting of internal medicine physicians/geriatricians, physiotherapists or occupational therapists, dieticians and nurses.

### Who will take informed consent? {26a}

Written and verbal information regarding the study will be given by the surgeon during the patient’s first visit at the outpatient clinic. A study nurse will have a follow-up telephone call and ask regarding participation. If a patient chooses to participate, an informed consent will be filled out at the day of interventions and obtained from one of the surgeons at the clinic. Eligible patients declining participation will be registered in a log. Written patient information and informed consent documents, in English versions, are attached to this article as Additional files 1 and 2.

### Additional consent provisions for collection and use of participant data and biological specimens {26b}

No additional data or biological specimens will be obtained.

## Interventions

### Explanation for the choice of comparators {6b}

The control group is treated according to usual care within an ERAS concept. Patients in the control group are assessed through some of the instruments that are used for the intervention group. These measurements are conducted by the regular ward staff (in general a nurse working at the surgery ward/outpatient clinic) at admission to hospital prior to surgery and at the postoperative outpatient visit (Table 2). These extra measurements are the only differences between the control group and patients not included in the trial.

### Intervention description {11a}

#### Standard protocol

Standard protocol for the treatment of colorectal cancer patients is according to best known practice, based on Swedish colorectal guidelines and within an ERAS setting. The preoperative investigation includes colonoscopy with biopsy (if possible), sometimes in combination with CT colonography, CT scans of the thorax and abdomen and in the case of rectal cancer also MRI of the lower abdomen. Each patient is thereafter discussed at a multidisciplinary team conference (MDT) and given a recommendation of treatment based on the endoscopic, radiologic and histopathologic findings. If the recommendation is surgery with a curative intent, this is scheduled within 4 weeks from their first visit at the outpatient clinic. According to the Swedish standardized referral pathways for colorectal cancer [36], the aim is to proceed from suspicion of colorectal cancer to surgery within 39 days. During this time, each patient has another appointment with the surgeon, followed by a pre-anaesthetic assessment by an anaesthesiologist. Interdisciplinary referrals (e.g. to cardiologist, lung physician) and/or further radiologic and physiologic examinations (e.g. UCG, spirometry) are made if deemed necessary through these evaluations. The patients are admitted to the surgical ward on the day before the planned surgery or the day of the operation. All surgical specimens are analysed by a pathologist and the postoperative TNM stage is determined. When the postoperative staging is completed, all patients are again discussed at MDT and a recommendation is made regarding following oncological treatment and future follow-up. All patients have a scheduled visit at the outpatient clinic in approximately 2 months post-surgery, to receive pTNM information, evaluate the postoperative recovery and plan for any following treatment and follow-up.

#### Intervention group

**Fig. 1:**
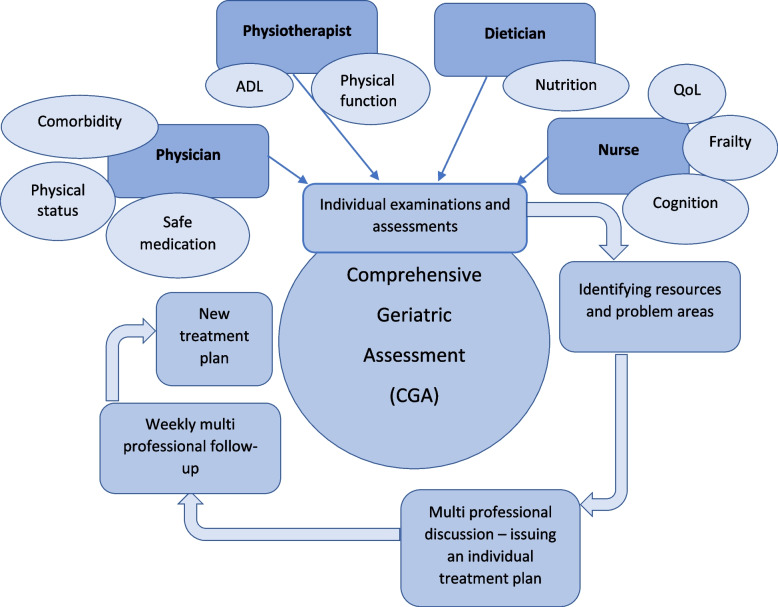
Description of the CGA and care concept in the study

The patients in the intervention group receive standard care according to the description above but with the CGA and care intervention in addition. As soon as a patient has been randomized to the intervention group, they are scheduled for an “intervention day”, which takes place on pre-decided dates (once every week). During this day, each patient has an individual meeting with an internal medicine physician with geriatric profiling, a study nurse, a physiotherapist or occupational therapist and a dietician. The team conducting the screenings receives introduction about the intervention and education regarding the CGA concept prior to entering the study, and they are also able to address the local expertise of CGA if questions arise during the evaluations. Each profession evaluates the patient clinically and according to the determined screening tools and issues an individual action plan based on these results (Fig. 1). The recommendations are individually prescribed and based on the health care professional’s evaluations based on the standardized screening, according to the CRF. The instruments used by each profession at different measurement points are stated below (Table 1).

**Table 1:** Screening instruments and assessments used for the intervention group, at baseline, at post-op visit (approximately 2 months post-op) and at 12 months post-op

Health care professional	Outcome and instruments	Other evaluations	Time of measurements
Internal medicine physician	- **Comorbidity**—*Charlson comorbidity index (CCI)* - **Medication safety**	- Review of SMA and current medications - Overall assessment of physiological status (including blood pressure and ECG)	**CCI** - Baseline - Post-op visit
Study nurse	- **Frailty**—*CFS-9* - **HRQoL**—*EQ-5D-5L* - **Cognition**—*MMSE* - **Medication safety**—*SMA and list of medications* -** Complications**—*Clavien-Dindo scale*		**CFS-9** - Baseline - Post-op visit **EQ-5D-5L** - Baseline - Post-op visit - 12 months post-op **MMSE** - Baseline **SMA** and list of medications - Baseline - Post-op visit **ADL** - Post-op visit **Complications** - Post-op visit
Physiotherapist	- **Dependence**—*ADL-step* - **Physical function** *hand grip strength (HGS) and six-minute walk test (6MWT)*	Overall assessment of the physical status	**ADL** - Baseline **HGS** - Baseline **6MWT** - Baseline
Dietician	- **Nutrition**—*MNA-SF*	Overall assessment of the nutritional status	**MNA-SF** - Baseline - Day of/day before surgery

All patients in the intervention group are discussed weekly in a group consisting of at least one representative from each profession. During these weekly meetings, the expected benefit of the interventions is weighed against the risk of delaying the surgery in each individual case. Adherence to the individually prescribed recommendations is evaluated and when the intervention is completed, or the patient for any given reason is not able to follow the recommendations, the intervention is terminated. To allow the CGA and care intervention to reach as much effect as possible, we have argued the need of, in some cases, allowing the time from diagnosis to surgery to be somewhat prolonged. In general, that time frame is 2–6 weeks but may in the trial setting be extended for up to 8 weeks. The overall objective is though, as in conventional care, to prepare the patient as quickly as possible for surgery without taking unnecessary medical risks. The postoperative phase is similar in both groups (Fig. 1).

#### Control group

The patients in the control group are assessed using some of the instruments that are used for the intervention group. These measurements are conducted by the regular ward staff (in general a nurse working at the surgery ward/outpatient clinic) at admission to hospital prior to surgery and at the postoperative outpatient visit (Table 2). These extra measurements are the only differences between the control group and patients not included in the trial treated according to standard protocol.

**Table 2:** Screening instruments used for the control group, at baseline, at post-op visit (approximately 2 months post-op) and at 12 months post-op

Health care professional	Outcome and instruments	Time of measurements
Ward nurse/nurse at the outpatient clinic	- **Frailty**—*CFS-9* - **HRQoL**—*EQ*-5D-5L - **Cognition**—*MiniMental State Examination (MMSE)* - **Medication safety**—*Safe Medication Assessment (SMA) and list of medications* -** Dependence**—*Activities of Daily Living (ADL-step)* - **Nutrition**—*Mini-Nutritional Assessment-Short Form (MNA-SF)*	**CFS-9** - Baseline - Post-op visit **EQ-5D-5L** - Baseline - Post-op visit - 12 months post-op **MMSE** - Baseline **SMA** and list of medications - Baseline - Post-op visit **ADL** - Baseline - Post-op visit **MNA-SF** - Baseline - Day of/day before surgery
Member of the project steering groups	- **Complications**—*Clavien-Dindo scale* - **Comorbidity**—*Charlson comorbidity index (CCI)*	**Complications** - Post-op visit **CCI** - Baseline - Post-op visit

#### Comprehensive geriatric assessment and care

The team conducting the CGA and care intervention in the trial consists of internal medicine physicians with geriatric profiling, study nurses, physiotherapists (and/or occupational therapists) and dieticians. The team works in close contact with the colorectal surgeons at the clinic. During the day of interventions, each patient is individually assessed by one member of each profession (Fig. 1).

The multimodal concept of comprehensive geriatric assessment and care is considered best practice when treating frail elderly patients [30, 37]. The multiprofessional and team-based examinations and assessments of the elderly individual identify both problems and resources in the elderly population and the assessments should lay the grounds for recommended treatment and follow-up. The use of CGA enables underlying unidentified conditions and problems to be identified and, if needed, specifically targeted, optimized and treated [38, 39]. Considering sarcopenia and nutritional problems being highly present amongst the frail elderly, physiotherapists (and/or occupational therapists) and dieticians are important members of the CGA and care team. A geriatric assessment, especially with a focus on medications, including risks of polypharmacy, inappropriate medications and ADRs, is also crucial [30, 40, 41].

### Criteria for discontinuing or modifying allocated interventions {11b}

At the weekly follow-up where every patient currently in the intervention group is discussed, each profession describes the planned intervention and the progress since the last meeting. If indications arise that there are compliance issues or complications (e.g. bowel obstruction, bleeding) requiring more urgent treatment, or when the intervention is deemed not to have any further benefits, the intervention will be terminated, and the patient will proceed to surgery without further delay.

### Strategies to improve adherence to interventions {11c}

There is a limited number of health care professionals working for each profession in the trial, as one way of making the continuity to the study protocol as great as possible. Prior to the study start, the participating health care professionals underwent education in using the instruments and expertise on each assessment tool is available if needed.

The project steering group meets regularly and performs internal monitoring of entered forms and CRFs, to assure compliance with the protocol and to identify eventual errors.

Prior to the weekly follow-up meetings, representants of at least one health care profession participating in the CGA team have contact with the intervention patient in order to evaluate the intervention, e.g. if the patient is using the newly prescribed medications, following the exercise programme, etc.

### Relevant concomitant care permitted or prohibited during the trial {11d}

The inclusion of a patient in the trial does not alter standard protocol—the same investigations, appointments, treatments, etc. will be offered and carried out. The only amendment to standard protocol is the addition of CGA and care, to patients in the intervention group.

### Provisions for post-trial care {30}

There are no provisions for ancillary or post-trial care, and this care will not be any different to the standard protocol for patients included in the study. As in conventional care, patients are insured under the Swedish patient insurance (LÖF) and may be entitled to compensation under the Swedish Patient Injury Act.

### Outcomes {12}

Primary endpoint:

- All-cause 90-day mortality

Secondary endpoints:

- Length of hospital stay and the total number of hospital days within 3 months after discharge
- Discharge destination (home or nursing facility) and use of home help services
- 30-day readmission rates
- ADL performance at follow-up in comparison to baseline
- Safe medication assessment at follow-up in comparison to baseline, adverse drug reactions (ADRs), underuse of evidence-based drug therapy and effects of inappropriate medications (analysed retrospectively)
- CFS-9 score at follow-up in comparison to baseline
- Postoperative complications according to the Clavien-Dindo scale at follow-up
- Health-related quality of life (HRQoL) at follow-up in comparison to baseline
- Cost-effectiveness based on mortality and HRQoL data (quality-adjusted life years [QALYs]) and costs of hospital care and primary care at baseline, 2 and 12 months post-surgery
- All-cause 1-year mortality

#### Outcomes and measurements

- *Hospital stays and discharge destination*, data regarding the length of hospital stay, total number of hospital days within 3 months after discharge, discharge destination and use of home help services and 30-day readmission rates for all participants is collected from patient medical records.
- *Dependence*, evaluation of the level of independence/dependence in an elderly individual is of great importance for example when establishing the need of home help services or other assistance. This is assessed using the activities of daily living (ADL)-staircase, which is a validated and commonly used instrument. The screening results in a score from 0 to 9, where 0 is completely independent and 9 is completely dependent [42, 43]. A clinically significant change is defined as a change of minimum 1 step in any direction on the ADL-staircase. ADL-function is assessed at baseline and at post-op visit (approximately 2 months post-surgery). The patients are assessed by a physiotherapist or a study nurse at baseline and follow-up.
- *Physical function*, the physiotherapists are also using hand grip strength (HGS) and six-minute walk test (6MWT) to make an overall assessment of the patient’s physical status to establish individual recommendations on physical activity and exercise. Both HGS and 6MWT have previously been used in assessments of frail elderly patients [44, 45]. The tests are performed at baseline and not at follow-up and are only conducted on patients in the intervention group as part of the CGA and care intervention.
- *Nutrition*, malnutrition is common in the elderly population, especially if hospitalized, living in nursing facility or having home help services. Malnutrition is often unrecognized if not specifically targeted. The Mini-Nutritional Assessment-Short Form (MNA-SF) is a validated instrument for nutritional screening in frail elderly individuals. It renders a score which places the patients in one of three groups: normal nutritional status, at risk of malnutrition or malnourished [46, 47]. A clinically significant change is defined as a change in group classification. The patients in the intervention group are screened by a dietician at baseline and by a ward nurse or a dietician at the day of/day before surgery. The patients in the control group are screened by a study nurse at both measurements.
- *Medication safety*, polypharmacy and the use of potentially inappropriate medications is a growing issue in the elderly population. There is often a need of reviewing and altering an elderly individual’s prescribed medications to reduce the risk of adverse drug reactions and to enable beneficial pharmacological treatments [48–50]. The Safe Medication Assessment (SMA), including a review of the current list of medications [51], is used as a basis for assessing the individual patient’s medications in the trial. The instrument renders a score of 0–30, where a higher score indicates a safer treatment. A clinically significant change is defined as a shift of minimum 1 point in either direction. The questionnaire is filled out by a study nurse at baseline and at revisit, for all participants. To identify adverse drug reactions (ADRs), underuse of evidence-based drug therapy and effects of inappropriate medications, scoring scales [49, 50] and clinical judgements based on medical records will be used. These judgements will be retrospectively performed by senior clinicians [52].
- *Cognition*, a decline in cognitive function is naturally present in the ageing process. Some elderly individuals are experiencing a more pronounced decline, resulting in cognitive impairment or dementia [53]. In the trial, the Mini-Mental State Examination (MMSE) is used as a screening instrument of cognitive function at baseline. It is a validated and reliable assessment tool in screening for cognitive impairment [54]. The screening is performed by a study nurse at baseline for all participants, but the measurement is not done at follow-up.
- *Frailty* is an independent risk factor of adverse health outcomes and death [21] and also a predictor of increased hospital and health care costs [25]. The degree of frailty is assessed using the Clinical Frailty Scale (CFS-9). The scale ranges from 1, “very fit”, to 9, “terminally ill”. The scale has been validated and proven to be of prognostic value in clinical practice [26, 55, 56]. The estimates are performed by a study nurse at baseline and at revisit, for all participants. A clinically relevant change is defined as a shift of minimum 1 step on the scale. During the initial phase of the trial’s recruitment, a revision of the CFS-9 scale was made, including minor adjustments in the description of the different levels of frailty [57]. Before the updated version (v 2.0) was put into use, a new application to the Swedish Ethical Review Authority was made and was approved on 2021-05-31.
- *Complications*, postoperative complications are common in elderly patients undergoing colorectal cancer surgery. The complications can result in additional treatments, reoperations, the need of intensive care and death [7, 8]. The Clavien-Dindo classification is a validated and clinically relevant instrument for identifying and grading adverse postoperative events [58]. The postoperative complications are assessed by a physician based on data from the medical records at the time of revisit, for all participants.
- *Comorbidity*, the patient’s comorbidity is valued through the Charlson comorbidity index (CCI). The instrument is used to assess the total burden of disease and predicts a 10-year mortality for a patient, by using 19 categories of comorbidities. Each condition is given a grade of 1, 2, 3 or 6, based on the risk of death associated with the specific comorbidity [59, 60]. The CCI index estimate will be performed by a physician, based on data from the medical records at baseline and at revisit, for all participants.
- *Health-related quality of life (HRQoL)*. The instrument EuroQoL-5D-5L (EQ-5D-5L) is used to assess HRQoL in the trial. The tool consists of a questionnaire containing five health dimensions with five response levels and of a visual analogue scale (VAS) where the patient estimates their own overall current health. Each estimated score is represented by an index value which is derived from a pre-defined value set [61, 62]. The assessments are overseen by a study nurse at baseline and at follow-up (2 and 12 months), for all participants.
- *Health economical calculations*. A health care system perspective will be applied including hospital and primary care. Cost-effectiveness will be analysed based on mortality and HRQoL data (quality-adjusted life years [QALYs]) and costs of hospital care and primary care at baseline, 2 and 12 months post-surgery. An incremental cost-effectiveness ratio (ICER) is calculated regarding cost/QALY gained. QALYs are calculated by multiplying the health state value with the patient’s survival time at 12 months; see also the “Statistical methods for primary and secondary outcomes” section. Data regarding health care costs will be collected and documented for all participants at 2 months and 12 months from baseline. Hospital costs will be obtained from the Swedish Cost-per-Patient database [63] and from hospital-specific administrative systems. Primary health care costs will be collected from the administrative regional health care database in Västra Götalandregion (VEGA) [64] and by the use of models and actual health care contacts.
- *Mortality*, mortality data will be extracted from patient medical records and cross-checked against the Swedish Population Registry regarding all participants at 2, 3 and 12 months from baseline.

### Participant timeline {13}

**Fig. 2:**
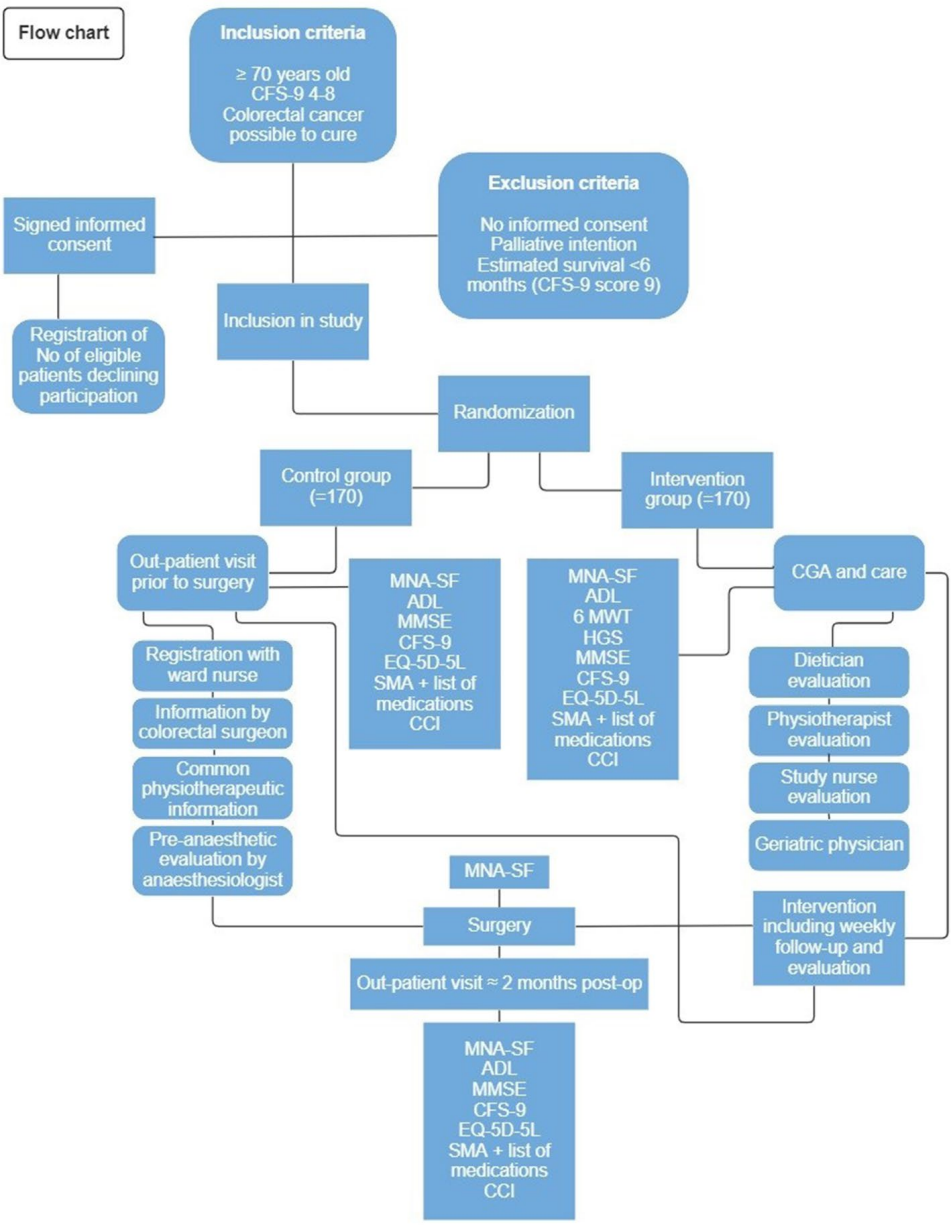
Flowchart of the patient flow through the trial. Displaying the differences between control and intervention groups and the screening instrument used at different measuring points

The flowchart of the time schedule, interventions, assessments and visits for participants is shown in Fig. 2.

### Sample size {14}

In earlier studies of elderly frail patients undergoing colorectal surgery, 90-day mortality has been reported between 5 and 20% in the frail group and 0–7% in the non-frail group [6, 13]. One-year mortality has been reported to be above 20% in the frail group and up to 10% in the non-frail group [6, 14]. To prove a 10% absolute reduction in 90-day mortality from 15 to 5% in the intervention group, 170 patients must be included in each group (power 80% and significance level of 0.05). The expected loss to follow-up is 5% which has been compensated for in the sample size.

### Recruitment {15}

The intention is to include 340 patients, 170 in the intervention group and 170 in the control group. All patients aged ≥ 70 with a recently diagnosed cancer of the colon or rectum, available for curatively intended surgery, are recognized at the weekly MDT. At their next-coming appointment at the outpatient clinic, they are screened for frailty using the CFS-9 instrument. Thereafter, they receive verbal and written information regarding the study and are offered inclusion if they meet all inclusion criteria and no exclusion criteria. The recruitment of patients is on-going, and at present, approximately 50 patients have been included in the trial. The inclusion rate has been lower than anticipated, and we assume this is partly due to the COVID-19 pandemic and its impact on the workload for the health care staff and patients’ care seeking behaviours. As the trial moves forward and further centres are added, we predict the inclusion rate will increase. We assume the recruitment period will continue for another 2 years.

Since all patients undergoing surgery for colorectal cancer are discussed at MDT in accordance with the Swedish standardized care procedures for colorectal cancer, we will be able to identify all eligible patients and continue with frailty screening.

## Assignment of interventions: allocation

### Sequence generation {16a}

The allocation sequence is generated by the project leader manually in blocks of 10 (intervention/control). The envelopes are mixed, then numbered and drawn in numerical order as patients are included. The randomization is stratified for colon or rectal cancer.

### Concealment mechanism {16b}

Patients are randomized to the control or intervention group. The randomization is performed in the outpatient clinic, using sequentially numbered, thick, opaque sealed envelopes.

### Implementation {16c}

Enrolment of participants is made by the surgeon. Assignment of participants to interventions is made by a study nurse or local principal investigator.

## Assignment of interventions: blinding

### Who will be blinded {17a}

Neither participants nor caregivers are blinded to group assignment, as the intervention will be apparent. However, results will be analysed without knowledge of details of the groups.

### Procedure for unblinding if needed {17b}

Not applicable, there is no blinding.

## Data collection and management

### Plans for assessment and collection of outcomes {18a}

Data regarding the following outcomes: length of hospital stay and total number of hospital days, discharge destination, 30-day readmission rates, ADL performance, safe medication assessment and medication lists, CFS-9 score, postoperative complications and HRQoL are documented on the CRFs at baseline and at the postoperative visit. Data regarding mortality at 3 and 12 months will be collected and documented separately. Data needed for the health economical calculations and cost-effectiveness analysis; health care costs at 2 months and 12 months, HRQoL at baseline, 2 and 12 months follow-up and mortality data at 2 and 12 months will be collected and documented separately. A more detailed description of the data collection and the study instruments, along with their reliability and validity, are found under the “Outcomes {12}” section. The CRFs used for the control versus intervention group, in English versions, are attached to this article as Additional files 3 and 4.

### Plans to promote participant retention and complete follow-up {18b}

See items 11b and 11c. The follow-up in the study is performed at the same time as the postoperative follow-up regarding the cancer disease. Participants who choose to discontinue their participation in the trial will be registered in a log and no further measurements or registrations will be made. Participants who deviate from the protocol or fail to appear at follow-up will be checked against the mortality registry but not further registered.

### Data management {19}

Each participant will be dealt a study number which further is used for identification. A specific key file is created for the identification, where personal identity numbers and study numbers are stored. This key file will only be kept at a hospital computer belonging to the local principal investigator. All examination results and medical considerations are documented in the medical chart. This is protected by the Swedish Law of Secrecy and Public Access. A CRF for each patient and all entered screening instruments (Tables 1 and 2) are obtained and held in a project-specific database and in patient-specific study binders.

There is no double data entry. Members of the steering committee intermittently conduct internal monitoring where the quality of entered study data is assessed. Likewise, an assessment of adherence to the protocol is conducted during these meetings. Adherence to the individual recommendations made by the health care professionals during the intervention is assessed during the weekly follow-up meetings (see item 11c).

### Confidentiality {27}

The key file is kept at a hospital computer belonging to the local principal investigator who is responsible for the collected personal data. The information is kept in accordance to the EU Data Protection Regulation. The information will only be shared with the project steering group. The information will be kept for 10 years and will not be disclosed to a third party. Processing of personal data will be done according to GDPR and in concordance with the Swedish Law of Secrecy and Public Access. Participants can, at any time, request their data to be retracted from the database. Likewise, participants are authorized to correct, delete or restrict the use of their information, at any time.

### Plans for collection, laboratory evaluation and storage of biological specimens for genetic or molecular analysis in this trial/future use {33}

Not applicable, no biological specimens will be kept in the trial.

## Statistical methods

### Statistical methods for primary and secondary outcomes {20a}

The primary analysis will be performed according to “intention to treat”. As a secondary analysis, a per-protocol analysis will be made. The dataset will be closed, an analysis plan will be made and a multivariate analysis with relevant confounding factors will be performed.

QALYs are calculated by multiplying the health care state with the patient’s survival time at 12 months. The HRQoL is considered linear between 2 values (index and 12-month follow-up). If a person dies during follow-up, the HRQoL at index is carried forward to the time of death. If only HRQoL at index is available (missing data at the 12-month follow-up), the score is carried forward, and, conversely, if only the 12-month follow-up score is available (i.e. data missing at index), this score is carried backward to the index. If a person dies during the index care episode, the QALY is set to 0. Adjusted data regarding costs and effects might be used in the cost-effectiveness analysis. Adjustments are done for age, sex and CCI.

### Interim analyses {21b}

There are no planned interim analyses or stopping guidelines.

### Methods for additional analyses (e.g. subgroup analyses) {20b}

Data will be analysed according to the Intention-to-treat (ITT) principle. Subgroup analyses and sensitivity analyses are planned according to secondary aims, as are adjusted analyses using multiple logistic regression/Cox regression.

### Methods in analysis to handle protocol non-adherence and any statistical methods to handle missing data {20c}

The intention-to-treat (ITT) principle will be applied.

### Plans to give access to the full protocol, participant-level data and statistical code {31c}

The study protocol is available at ClinicalTrials.gov and at researchweb.org/is/vgr. The datasets analysed during the current study and statistical code are available from the corresponding author on reasonable request.

## Oversight and monitoring

### Composition of the coordinating Centre and trial steering committee {5d}

The trial has no separate steering committee, the trialists, Mattias Prytz (principal investigator), Niklas Ekerstad and Eva Angenete, act as the steering committee.

### Composition of the data monitoring committee, its role and reporting structure {21a}

A data monitoring committee was not considered as this is regarded a low-risk intervention.

### Adverse event reporting and harms {22}

Any study-related adverse events will be reported to the Swedish Health and Social Care Inspectorate, as in conventional care.

### Frequency and plans for auditing trial conduct {23}

The trialists at each participating centre have local internal monitoring meetings at a minimum of twice per year. There are also joint follow-up meetings of the currently included centres once per term.

### Plans for communicating important protocol amendments to relevant parties (e.g. trial participants, ethical committees) {25}

Any changes made to the protocol will be amended and communicated to the Ethics board. The changes will also be included in the clinical trial register (https://clinicaltrials.gov/) and updated at https://researchweb.org/is/vgr.

## Dissemination plans {31a}

Results from the RCT will be disseminated through presentations and publications. Manuscripts will be written by the study group members and affiliated researchers.

## Discussion

A majority of colorectal cancer patients are elderly, and in most cases, the treatment objective is to provide the same curative therapy as for younger patients. There have been significant improvements in the management of colorectal cancer in the elderly during recent years. Though, there is a growing interest in further optimizing the process, to decrease the higher mortality and complication rates, especially in the immediate postoperative phase. In other medical circumstances, an individualized plan and treatment according to CGA and care has improved outcomes for the group frail elderly. We know that frail elderly suffers higher risks of postoperative complications and mortality after colorectal cancer surgery and there is circumstantial evidence pointing in the direction of positive effects of CGA and care also for patients with colorectal cancer. However, there is no randomized intervention study of substantial size and quality to support this assumption.

Arguing for stretching the time span from diagnosis and treatment decision to surgery up to 8 weeks poses some ethical and practical concerns. Patients may be reluctant to participate in the study due to fear of progressive disease during the time until surgery. It may also be a larger psychological stress knowing that the cancer is left untreated for a longer time. However, our hypothesis is that the positive effect of the individualized CGA and care intervention on postoperative complications and mortality will outweigh the possible—but not scientifically shown—enhanced risk of progressive disease due to delayed treatment of the tumour. There is however no evidence in the literature that a delay of treatment of this timespan would have a negative effect on overall survival or disease-free survival [65–68]. There are rather indications that optimizing a patient’s medical situation preoperatively can improve outcomes after surgery, even if the time until surgery is somewhat extended. We have argued this in our application to the Ethics board and have gained approval from the Swedish Ethical Review Authority for this regimen. Also, the intervention team is in every case focused on keeping the intervention as short and effective as possible. As soon as the intervention does not provide further use or when the maximum time of 8 weeks is reached, the patient proceeds to surgery without further delay.

The study is set up as a multicentre trial. There is a risk that routines differ between hospitals which could require deviations from the protocol. The CRFs and assessment tools are the same at every hospital which forces coherence to the most important variables of the study. Likewise, all participating hospitals are following the ERAS concept and the guidelines stipulated in the standardized care process of colorectal cancer published by the National Board of Health and Welfare in Sweden. This further strengthens the accordance between centres.

Another risk of conducting a randomized controlled trial is that the novel ideas and processes of the intervention could affect the way the health care professionals treat patients in the control group. Since the trial brings notice to the fact that frail elderly is a group at particular risk of adverse effects, it is possible that the staff at the clinic will treat participating patients in a different way, e.g. pay extra attention to symptoms suggesting postoperative complications or becoming more aware of perioperative nutrition problems etc. This could affect, and possibly, diminish the results from the study. To assure that the control group is treated according to standard clinical praxis, we have tried to separate the two groups as much as possible prior to surgery. The intention is to schedule the day of interventions, when the patients in the intervention group meet the CGA team, on a day where patients in the control group are not visiting the hospital. The health care professionals in the CGA team will not treat the control group during their stay at the hospital, in connection to their surgery.

We believe this trial could bring important light to the group of frail elderly in terms of surgery, primarily of colorectal cancer. It is clearly shown that this group is suffering poorer outcome after surgery, which also generates higher health care costs. If a CGA and individualized care treatment could decrease postoperative complication and mortality rates, as well as being cost-effective, it would be of great value in future care of frail elderly patients with colorectal cancer.

## Trial status

Recruitment began on 2020-10-01 in Northern Älvsborg County Hospital (NÄL), part of the NU-Hospital Group in Trollhättan, Sweden. Participation of the second study centre, Östra Hospital, Sahlgrenska University, in Gothenburg is estimated to begin in autumn 2021. Protocol version 8.1, June 2021. Recruitment is estimated to be completed approximately 2023-12-31.

## Supplementary Information

**Additional file 1.** Patient information.

**Additional file 2.** Patient informed consent form.

**Additional file 3.** CRF control group.

**Additional file 4.** CRF intervention group.

## Data Availability

The final dataset will be kept by the principal investigator and can be supplied on reasonable request. The information will be kept for 10 years and will not be disclosed to a third party.

## Abbreviations

6MWT: Six-minute walk test
ADL: Activities of daily living
ADR: Adverse drug reaction
CCI: Charlson Comorbidity Index
CFS-9: Clinical Frailty Scale
CGA: Comprehensive geriatric assessment
CRC: Colorectal cancer
CRF: Case report form
CSHA: The Canadian Study of Health and Ageing
ERAS: Enhanced recovery after surgery
GDPR: General Data Protection Regulation
HGS: Hand grip strength
HRQoL: Health-related quality of life
ICER: Incremental cost-effectiveness ratio
ITT: Intention to treat
MDT: Multidisciplinary team conference
MMSE: Mini-Mental State Examination
MNA-SF: Mini-Nutritional Assessment-Short Form
QALY: Quality-adjusted life years
RCT: Randomized controlled trial
SCRCR: Swedish Colorectal Cancer Registry
SMA: Safe Medication Assessment
