# Supplementary material for: Effect of comprehensive geriatric assessment for frail elderly patients operated for colorectal cancer—the colorectal cancer frailty study: study protocol for a randomized, controlled, multicentre trial
Source: Trials. 2022 Nov 17;23:948. doi: 10.1186/s13063-022-06883-9 (PMC9670054; doi:10.1186/s13063-022-06883-9)
Supplement: Supplementary file 1 — Additional file 1. Patient information. [file 13063_2022_6883_MOESM1_ESM.pdf]

Region Västra Götaland

2020-11-06

Patient information regarding research project:

”Effect of CGA and care for frail elderly patients operated for colorectal cancer – The CRC Frailty study"

Swedish title ”Kan preoperativ övergripande geriatrisk bedömning och vård reducera mortaliteten efter operation för tjock- och ändtarmscancer hos sköra äldre?”

Background and aim

Colorectal cancer is the third most common type of cancer in Sweden. Each year approximately 6000 individuals gets diagnosed. Out of these, 65 % are older than 65 years. When curative treatment is possible, surgery is the treatment of choice, sometimes in combination with radiation- and/or chemotherapy.

As life expectancy is increasing, research regarding the elderly population and surgery is important and of rising interest. Age by its own is a rough instrument when estimating risks in regards of surgery. Research has shown that by using different screening tools one is possible to estimate the degree of frailty, which is an independent risk factor for prognosing outcome in several different conditions and treatments. This correlation is true also with colorectal cancer and it is a fact that serious complications after surgery is increased in frail patients 70 years or older.

In other areas, e.g. regarding surgery for hip fracture, it has been shown that specific evaluation of elderly frail patients (so called Comprehensive Geriatric Assessment – CGA), including evaluation of physiotherapist, dietician and thereafter targeted treatments improves outcome after surgery. If this is the case also with elderly frail patients undergoing surgery to cure colorectal cancer is not known, since it has not been studied earlier.

Present study

We are conducting a study where all patients 70 years or older who are going through a planned surgery to cure colorectal cancer are offered inclusion. The aim is to investigate whether an individualized assessment and care of frail

elderly patients prior to surgery for colorectal cancer can improve outcome post-surgery.

If you accept participation and reach the criteria for frailty (score 5-8 on CFS-9 scale), a randomization procedure will decide if you are to enter the intervention group (CGA and care in addition to standard care) or the control group (standard care). If participating in the study, you will undergo pre-operative assessment and surgery in the precise manner as if you were not part of the study. The intervention group will in addition to this also take part of the individually tailored geriatric assessment, performed by a geriatric physician, a physiotherapist and a dietician. With base from these evaluations, individual recommendations and treatments will be placed.

If the CGA concludes a need of optimization prior to surgery, we will allow a somewhat prolonged time from diagnosis to surgery – at most 8 weeks. This has carefully been considered in the study planning and the possible benefit of these individual acts is believed to overweigh the risk of, in certain cases, delay surgery.

Participation in the study is completely voluntary and you can at any point decide to leave the study, without having to motivate your decision or give any explanation. If you decline entering the study or chose to abort your participation this will not affect your treatment.

Data management and confidentiality

We will, as always, record all significant facts in your medical charts. This is protected by the Swedish Law of Secrecy and Public Access. Study specific information will be coded and transferred to a project specific database. Each participant will be dealt a study number which further will be used for identification. A specific key file will be created for the identification, where social security numbers and study numbers are stored. This key file will only be kept at a hospital computer belonging to the principal investigation, Dr Prytz. Dr Prytz is also responsible for the collected personal data. The information will be kept for ten years. The data that will be kept in the database is: your diagnosis, age, gender, score on CFS-9, ADL-function, for how many days you are admitted to hospital, what kind of housing situation your discharge destination is and if it arises any medical complications during and after your surgery.

We will not be sampling any biological material. Your information will be kept in accordance with the EU Data Protection Regulation. We will not disclose any of your information to a third party. You can, at any time, request your data from the project database. You also have the right of correcting, deleting or restricting the use of your information. If you wish to do any of the above, contact the principle investigator, Dr Prytz. You are also entitled to enter complaints to the Data Inspectorate. The data protection officer of NU Hospital Group is Niklas

Claesson, reached at 010-435 65 26 or niklas.claesson@vgregion.se. The results from the study will be presented as scientific articles.

Responsible for the study are Chief Physician Mattias Prytz and Resident Physician Maria Normann at the department of surgery, NU Hospital Group and Chief Physician Niklas Ekerstad, cardiologist and internist.

If you have any further questions you can get in contact with us at: 010-435 34 00 during office hours.
