# Supplementary material for: Effect of comprehensive geriatric assessment for frail elderly patients operated for colorectal cancer—the colorectal cancer frailty study: study protocol for a randomized, controlled, multicentre trial
Source: Trials. 2022 Nov 17;23:948. doi: 10.1186/s13063-022-06883-9 (PMC9670054; doi:10.1186/s13063-022-06883-9)
Supplement: Supplementary file 2 — Additional file 2. Patient informed consent form. [file 13063_2022_6883_MOESM2_ESM.pdf]

Region Västra Götaland

2020-11-06

Informed consent regarding participation in research study:

”Effect of CGA and care for frail elderly patients operated for colorectal cancer – The CRC Frailty study”

The department of surgery is at present conducting a research project studying the use of multidisciplinary assessment and care of frail elderly prior to planned surgery for colorectal cancer. The aim is to evaluate if this assessment and care, regarding the medical situation, physical abilities and nutritional status could improve outcome post-surgery.

Part taking in the study is voluntarily and the consent can at any time, and with immediate effect, be withdrawn. Processing of personal data will be done according to GDPR and in concordance with the Swedish Law of Secrecy and Public Access.

I have received verbal and written information regarding the study. I accept all conditions above and hereby approve to my coded medical charts being transferred to the project database and used in the purpose of research in the above described project.

Place and date, single-handedly entered by the participant

____

Date, place

____

Signature

____

Name clarification and social security number

I have explained the study and have today received the patients consent to participate in the study

____

Date

____
Signature

____
Name clarification

Principal investigator is Chief Physician Mattias Prytz at the Department of Surgery NU Hospital Group. Contact: 010-435 34 00.
