# Supplementary material for: Effect of comprehensive geriatric assessment for frail elderly patients operated for colorectal cancer—the colorectal cancer frailty study: study protocol for a randomized, controlled, multicentre trial
Source: Trials. 2022 Nov 17;23:948. doi: 10.1186/s13063-022-06883-9 (PMC9670054; doi:10.1186/s13063-022-06883-9)
Supplement: Supplementary file 3 — Additional file 3. CRF control group. [file 13063_2022_6883_MOESM3_ESM.pdf]

Frailty study
Control group

PATIENT DATA

Background facts – filled out at inclusion

Signed informed consent ☐

Age (digits): ________________

Gender: ☐ Female ☐ Male

Score CFS-9 (digits):_________________
(5-8 to be included in study)

Housing situation

☐ Own housing without home help services or home nursing

☐ Own housing with home help services

☐ Own housing with home nursing

☐ Living in nursing facility

☐ Other:______________________

____

Biochemical markers:

Hb (g/L): ___________________

Creatinine (μmol/L):____________

eGFR (ml/min/1,73m^3):_________

Diagnosis

☐ Colon cancer

☐ Rectal cancer

Staging according to cTNM

T_________N________M_______

Date of MDT

____

Date of first visit____________________________

Date of inclusion in study

____

Screening according to ERAS

Modified SGA	☐ A	☐ B	☐ C
Alcohol	☐ AUDIT-C (**attach form**)		
	☐ Score_______________		
Smoking	☐ No	☐ Ex-smoker	
	☐ Yes, number of pack-years_______________		

Signature

____

Frailty study
Control group

PATIENT DATA

Check list – filled out by study nurse during visit prior to admission to hospital

Conducted screenings (attach forms)

EQ-5D-5L	☐
MMSE	☐
SMA + list of medications	☐
MNA-SF	☐
ADL	☐
CCI	☐
CFS-9 (first page)	☐

Signature

____

Frailty study
Control group

PATIENT DATA

Filled out at admission to surgical ward

Date of hospital admission:______________________________

Date of surgery: ______________________________

ASA-classification: _____________

Screening (attach form)

MNA-SF: ☐

Signature

____

Frailty study
Control group

PATIENT DATA

Date of revisit: ___________________________
Score CFS-9 (digit):________________________________
Performed surgery:

☐ Resection of colon

☐ Right hemicolectomy

☐ Left hemicolectomy

☐ Resection of sigmoid colon

☐ Rectal resection

☐ Rectum amputation

☐ Other surgery, which:

____

Anastomosis/stoma (tick all appliable)

☐ Primary anastomosis

☐ Temporary stoma

☐ Permanent stoma

Surgical method

☐ Laparoscopic surgery

☐ Laparoscopic surgery converted to open surgery

☐ Robot assisted surgery

☐ Open surgery

Staging according to pTNM:

T_______N_______M_______

Follow-up – filled out at revisit 8 weeks post-op

Screenings (attach forms)

EQ-5D-5L	☐
ADL	☐
SMA + list of medications	☐
CCI	☐
CFS-9	☐
MNA-SF	☐

Length of hospital stay in connection to surgery (dates):_______________________

Readmission within 30 days post-op:

☐ Yes – how many times:______________

☐ No

Total days of hospital stay, including in connection to surgery
(dates):_____________________________________

Discharge destination:

☐ Own housing without home help services or home nursing

☐ Own housing with home help services

☐ Own housing with home nursing

☐ Nursing facility

☐ Other:_________________________

____

Signature

____

Frailty study
Control group

PATIENT DATA

Post-op complication has arisen that:

☐ 1) is pharmacological treated with: antiemetics, antipyretics, analgesics, diuretics and/or electrolytes.

☐ 2) is treated with pharmacotherapy not mentioned above (eg blood transfusion or TPN)

☐ 3a) requires surgical, endoscopic or radiological intervention

☐ 3b) requires surgical, endoscopic or radiological intervention in general anaesthesia

☐ 4a) requires ICU care due to single organ failure

☐ 4b) requires ICU care due to multiorgan failure

☐ 5) lead to patient loss of life

□ **No noted complications**

Description of arisen

complication:______________________________________

____

____

____

____

____

Data from the first 12 months regarding mortality, health care costs and health-related quality of life (EQ-5D-5L) will be collected and documented separately from the CRF.

Signature

____
