# Supplementary material for: Effect of comprehensive geriatric assessment for frail elderly patients operated for colorectal cancer—the colorectal cancer frailty study: study protocol for a randomized, controlled, multicentre trial
Source: Trials. 2022 Nov 17;23:948. doi: 10.1186/s13063-022-06883-9 (PMC9670054; doi:10.1186/s13063-022-06883-9)
Supplement: Supplementary file 4 — Additional file 4. CRF intervention group. [file 13063_2022_6883_MOESM4_ESM.pdf]

Frailty study
Intervention group

PATIENT DATA

Background facts – filled out at inclusion

Signed informed consent ☐

Age (digits): ___________________

Gender: ☐ Female ☐ Male

Score CFS-9 (digits):____________________
(5-8 to be included in study)

Housing situation

☐ Own housing without home help services or home nursing

☐ Own housing with home help services

☐ Own housing with home nursing

☐ Living in nursing facility

☐ Other:_________________________

____

Biochemical markers:

Hb (g/L): ______________________

Creatinine (µmol/L):_____________

eGFR (ml/min/1,73m^3):__________

Diagnosis

☐ Colon cancer

☐ Rectal cancer

Staging according to cTNM

T__________N_________M________

Date of MDT ___________________________________

Date of first visit_________________________________

Date of inclusion in study

____

Screening according to ERAS

Modified SGA	☐ A	☐ B	☐ C
Alcohol	☐ AUDIT-C (**attach form**)		
	☐ Score_________________		
Smoking	☐ No	☐ Ex-smoker	
	☐ Yes, number of pack-years__________________		

Signature

____

PATIENT DATA

Frailty study
Intervention group

Check list – filled out by study nurse during the day of intervention

Date of day of intervention ____________________________

Conducted screenings (attach forms)

EQ-5D-5L	☐
MMSE	☐
SMA + list of medications	☐
MNA-SF	☐
ADL	☐
HGS	☐
6 MWT	☐
CCI	☐
CFS-9 (first page)	☐

Patient assessed by:

Study nurse	☐
Physician	☐
Physiotherapist/Occupational therapist	☐
Dietician	☐

Weekly interprofessional meeting:

Week 1 ☐ (date)__________

Continued intervention__________________

Week 2 ☐ (date)__________

Continued intervention__________________

Week 3 ☐ (date)__________

Continued intervention__________________

Week 4 ☐ (date)__________

Continued intervention__________________

Week 5 ☐ (date)__________

Continued intervention__________________

Week 6 ☐ (date)__________

Continued intervention__________________

Week 7 ☐ (date)__________

Continued intervention__________________

Week 8 ☐ (date)__________

Continued intervention__________________

Cause:______________________________

____

Signature

____

Frailty study
Intervention group

PATIENT DATA

Dietician

Screening

Evaluation according to MNA-SF

☐ Normal nutritional status

☐ At risk of malnutrition

☐ Malnourished

Further nutritional evaluations:____

____

Interventions

☐ Individual nutritional evaluation

☐ Enrichment products

☐ Dietary supplements

☐ Enteral nutrition

☐ Parenteral nutrition

☐ Other: _________________________

____

____

____

Follow-up

☐ Phone contact 1 (date)_____________

☐ Phone contact 2 (date)_____________

☐ Phone contact 3 (date)___________

☐ Food diary

☐ Weight and BMI

☐ Eating problems

Other: _____________________________

____

____

____

Signature

____

PATIENT DATA

Frailty study
Intervention group

Physiotherapist

Screening

ADL ☐

HGS ☐

6 MWT ☐

Further physiotherapeutic evaluations:____

____

Interventions

☐ History of every-day activities

☐ Information regarding physical activity and exercise

☐ Prescription of physical activity and exercise with referral

☐ Prescription of physical exercise program, linked in ExorLive

☐ Other intervention______________________

____

☐ No further intervention

Due to:___________________________________

____

Follow-up

☐ Contact by telephone 1
(date)__________________________________

☐ Contact by telephone 2
(date)__________________________________

☐ Contact by telephone 3
(date)__________________________________

☐ New screening/visit
(date)__________________________________

☐ Other:__________________________________

____

Signature

____

Frailty study
Intervention group

Physician

PATIENT DATA

1. Anamnesis ☐ (tick if performed)
   Current symtoms:____

____

____

2. Examination, blood pressure, lab works, ECG ☐ (tick if performed)

Blood pressure: _______________

ECG: ☐ Normal

☐ Pathologic

Comments:_____________________________

____

____

____

____

3. Review of medications including assessment of current list of medications ☐ (tick if performed)
   Comments:____

____

____

4. Evaluate the need of follow up ☐ (tick if performed)
   Comments:____

____

5. Other actions/comments:

____

____

____

____

Signature

____

Frailty study
Intervention group

PATIENT DATA

Filled out at admission to surgical ward

Date of hospital admission:______________________________

Date of surgery: ______________________________

ASA-classification: _____________

Screening (attach form)

MNA-SF: ☐

Signature

____

Frailty study
Intervention group

PATIENT DATA

Follow-up – filled out at revisit ca 8 weeks post-op

Date of revisit: ___________________________
Score CFS-9 (digit):________________________________
Performed surgery:

☐ Resection of colon

☐ Right hemicolectomy

☐ Left hemicolectomy

☐ Resection of sigmoid colon

☐ Rectal resection

☐ Rectum amputation

☐ Other surgery, which:

____

Anastomosis/stoma (tick all appliable)

☐ Primary anastomosis

☐ Temporary stoma

☐ Permanent stoma

Surgical method

☐ Laparoscopic surgery

☐ Laparoscopic surgery converted to open surgery

☐ Robot assisted surgery

☐ Open surgery

Staging according to pTNM:

T_______N_______M_______

Screenings (attach forms)

EQ-5D-5L	☐
ADL	☐
SMA + list of medications	☐
CCI	☐
CFS-9	☐
MNA-SF	☐

Length of hospital stay in connection to surgery (dates):_______________________

Readmission within 30 days post-op:

☐ Yes – how many times:______________

☐ No

Total days of hospital stay, including in connection to surgery
(dates):_____________________________________

Discharge destination:

☐ Own housing without home help services or home nursing

☐ Own housing with home help services

☐ Own housing with home nursing

☐ Nursing facility

☐ Other:_________________________

____

Signature

____

Frailty study

Intervention group

PATIENT DATA

Post-op complication has arisen that:

☐ 1) is pharmacological treated with: antiemetics, antipyretics, analgesics, diuretics and/or electrolytes.

☐ 2) is treated with pharmacotherapy not mentioned above (eg blood transfusion or TPN)

☐ 3a) requires surgical, endoscopic or radiological intervention

☐ 3b) requires surgical, endoscopic or radiological intervention in general anaesthesia

☐ 4a) requires ICU care due to single organ failure

☐ 4b) requires ICU care due to multiorgan failure

☐ 5) lead to patient loss of life

☐ **No noted complications**

Description of arisen complication:______________________________________

____

____

____

____

____

Data from the first 12 months regarding mortality, health care costs and health-related quality of life (EQ-5D-5L) will be collected and documented separately from the CRF.

Signature

____
